# Typhoid Fever Complicated with Broncho-Pneumonia

**Published:** 1865-12

**Authors:** N. S. Davis

**Affiliations:** Prof. Clinical Medicine, &c., &c.


### TYPHOID FEVER COMPLICATED WITH BRONCHO-PNEUMONIA.—CLINICAL CASES IN THE MEDICAL WARDS OF MERCY HOSPITAL. SUBSTANCE OF A CLINIC.

By N. S. DAVIS, M.D., Prof. Clinical Medicine, &c., &c.

GENTLEMEN:—A few days since, I directed your attention to two cases of typhoid or enteric fever in this ward, each complicated with serious pneumonic inflammation. One of them is still occupying the bed in which you saw him, towards the other end of the ward, and is now convalescent. He is a native of Norway, aged about 22 years, and had been sick ten or twelve days before his admission into the hospital.

At the time your attention was called to his case, he was presenting all the symptoms of the advanced state of typhoid fever, such as somnolency; muttering delirium; dry tongue and mouth; sordes on the lips and teeth; dryness of the skin, with congestion of cutaneous capillaries; tympanitic abdomen, with five or six thin, reddish-brown intestinal discharges daily. Upon this condition had rapidly supervened, during the three or four preceding days, short and hurried breathing, with loud bronchial ronchi over the whole anterior part of the chest, a mixture of dry and moist ronchi in the axillary and sub-axillary regions, and in the latter dulness on percussion. There was cough with a scanty expectoration tinged with dark blood, and the pulse was 120 per minute, small and weak. I then explained to you that the case presented all the phenomena of typhoid fever, with the enteric disease peculiar to that grade of fever well developed, on which had supervened universal congestion of the mucous membrane of the respiratory passages, with pneumonic infiltration of the middle lobes of both lungs, constituting a most dangerous condition, yet one frequently met with in this climate during the latter part of autumn and early spring.

You will remember that we directed a blister to be applied to his chest, while he took, internally, the emulsion of oil of turpentine and tincture of opium every four hours and one of the following powders between:—

℞. Pulv. Opii,---------------------------- 6 grs.
Tart. Ant. et Pot.--------------------- 1 gr.
Sacchar. Alba,-------------------------30 grs.

Mix, and divide into six powders.

Under that treatment he soon began slowly to improve. The symptoms of intestinal irritation first disappeared, and the ordinary anodyne cough mixture was substituted for the emulsion, and after four or five days the powders of opium and antimony were restricted to one every night. As already remarked, that patient is now convalescent. The other to whom I directed your attention, at the same clinic, and whose chest you had an opportunity of auscultating, was a case of typhoid fever, presenting complications of the same nature, but much more severe.

The patient, a German, aged about 25 years, instead of being somnolent, was affected with constant delirium and sub-sultus; the intestinal discharges were not only more frequent, but they were mixed with mucus and blood; and the whole right lung, from the clavicle to the diaphragm, yielded a complete dull sound on percussion, and only very slight respiratory or vesicular murmur by auscultation. At first, a blister was applied to the right side of the chest, and the same remedies given internally, as in the case previously alluded to. After continuing this treatment two full days, the intestinal discharges and abdominal tympanites were much improved, but the delirium, the muscular tremulousness, and the oppression of breathing were much increased. The pulse was small, soft, and frequent. Thinking it very desirable to lessen the nervous jactitation and delirium, so as to induce some sleep, and finding the powders of opium and antimony to fail, I caused them to be omitted and fifteen drops of chloroform given in their place every three hours, alternated with the turpentine emulsion. In twenty-four hours after commencing the use of the chloroform, his pulse and respirations were slower; the sub-sultus less; and he obtained short intervals of quiet sleep. The bowels also remained quiet. Consequently the same remedies were continued, only at a little longer intervals. On the afternoon of the second day, he not only remained more quiet and rational, with a fair amount of sleep, but he coughed less, and there was decidedly less dulness over the upper lobe of the right lung, with a corresponding increase of respiratory murmur. The bowels had also remained without any evacuation, and the abdomen only slightly tympanitic. The amount of chloroform now to be administered was reduced to ten drops every four hours, and an anodyne cough or expectorant mixture given instead of the emulsion. On the fourth day after the first exhibition of the chloroform, the patient appeared so much improved, in all respects, that I thought his convalescence had fairly begun. He was consequently ordered to have more nourishment, and the use of the chloroform restricted to ten drops each morning, noon, and evening. During the latter part of the following night, however, the patient suddenly expired. The death was preceded by no new symptoms sufficient to attract the attention of the nurses, and no *post mortem* examination was permitted. The immediate cause of the patient's death is, therefore, left to conjecture. It might have been syncope from some unobserved attempt to rise from the bed, or from the formation of emboli or fibrinous clots in the cavities of the heart. The very small quantity of chloroform which he had been taking during the preceding twenty-four hours, could hardly be suspected of having induced excessive anæsthesia of the nervous centres. We have administered that agent, in doses of from 10 to 15 drops, in several cases of typhoid fever with pneumonic complications, in which there was constant delirium and sleeplessness, and in all previous instances with the most satisfactory results.

The case now before you is a German by birth, aged about 25 years. He was admitted to the hospital three days since, and was reported to have been sick about two weeks previously. At my first visit after his admission, his face was suffused with a dark red flush; lips and tongue dry; expression dull; skin moderately hot and dry; pulse 112 per minute, small and weak; respirations short, and accompanied by coarse, dry, bronchial ronchi over both sides of the chest, intermixed with a sub-mucous rhonchus over the middle and lower part of the right lung, with considerable dulness, on percussion, over the latter region. There was also frequent cough, with but little expectoration. The mind was dull and more or less wandering, and slight hemorrhage from the nose. The abdomen was quite full and tympanitic, and he had been having from three to five thin, brown fœcal evacuations daily. You will recognize in this assemblage of symptoms, as well as in the present aspect of the patient before you, a well-marked typhoid condition, with the usual enteric disease of the aggregated glands, and, in addition, a dry, congested condition of the pulmonary mucous membrane, with considerable pneumonic engorgement of the middle and lower lobe of the right lung. To restrain the intestinal irritation and excessive evacuations, I directed him to have a teaspoonful of the turpentine and laudanum emulsion every four hours, and yesterday, finding the dry sounds still predominating in his chest, and the skin dry, I ordered one of the following powders to be taken between each of the doses of the emulsion:—

℞. Pulv. Opii,---------------------------- 8 grs.
Hydrarg. Chlorid. Mite,--------------- 6 grs.
Tart. Ant. et Pot.---------------------- 1 gr.
Sacchar. Alba,-----------------------20 grs.

Mix, divide into six powders.

The object of this was to induce such a change in the functions of the skin and pulmonary membranes as would result in free secretion from both, and thereby lessen the congested condition of the pulmonary capillaries, and the tendency to further infiltrations into the pulmonary textures. The patient has taken the powders just named, alternated with the emulsion, during the past twenty-four hours, and if you now step forward and examine him, you see his face less flushed, lips and tongue less dry, though there is still a dry crusted strip through the middle of the latter, and some sordes on his lips. The skin is relaxed and universally wet with warm perspiration. His countenance is still dull; mind somewhat wandering; and you see a little blood on the upper lip, indicating recent slight epistaxis. If you watch the motions of the chest, you see that the respirations are short, and too much abdominal. If you now take the stethescope and, one by one, listen to the anterior and right lateral part of his chest, you will perceive, still more clearly, the imperfect expansion of the chest by inspiration, while you find the dry, wheezing sounds of yesterday replaced with moist or mucous rhonchi, intermingled with only an occasional coarse, dry sound. If you move the stethescope to the anterior margin of the axillary region, you will hear less respiratory sounds of any kind, but if the patient articulates sounds, you will have decided vibration of voice, indicating increased density of that portion of thelung. Now, what is the exact pathological condition of our patient, and what the indications for further treatment?

The typhoid condition of the patient is strongly characterized; and though the general febrile condition is improved, as indicated by the soft and moist condition of the skin, the less tympanitic condition of the abdomen, and the more quiet condition of the bowels; yet the pulse is very soft, weak, and 115 per minute, the respirations are short, and the patient gives evidence of a feeling of exhaustion. He has evidently arrived at a critical period in his disease. He is vascillating to-day, between a tendency to convalesce on the one hand, and such a general depression of vital properties in his tissues as would soon cause a return of copious intestinal discharges, perhaps mixed with blood, with a rapid increase of infiltration into the pulmonary textures, followed by colliquative sweating, involuntary discharges and death.

Hence, it is necessary to adjust the further treatment with great care. If we continue the antimony and calomel in the powders, we shall increase the risk of the latter alternative. But if we can bring to our aid some agent calculated to give increased tone or contraction to the capillary system of vessels without restricting secretion, we may decide the progress of the case in favor of convalescence. With this view, we shall continue the turpentine and laudanum emulsion every four hours, and change the powders by substituting sulphate of quinine, 2 grs., and pulv. bloodroot, 1 gr., for the antimony and calomel in each powder, leaving the opium as before. As thus altered, one must be given half way between the doses of emulsion. Of course, in all such cases, the patient is carefully fed with animal broth, well-salted, and a porridge of sweet milk and wheat flour. The further progress of this case will be shown you at subsequent clinics.

NOTE.—This patient progressed well under the last prescription, and has since entirely recovered.

